# Is Every Wild Species a Rich Source of Disease Resistance? *Avena fatua* L.—Potential Donor of Resistance to Powdery Mildew

**DOI:** 10.3390/plants10030560

**Published:** 2021-03-16

**Authors:** Sylwia Okoń, Tomasz Ociepa, Aleksandra Nucia, Magdalena Cieplak, Krzysztof Kowalczyk

**Affiliations:** Institute of Plant Genetics, Breeding and Biotechnology, University of Life Science, 20-950 Lublin, Poland; tomasz.ociepa@up.lublin.pl (T.O.); aleksandra.nucia@up.lublin.pl (A.N.); magdalena.cieplak@up.lublin.pl (M.C.); krzysztof.kowalczyk@up.lublin.pl (K.K.)

**Keywords:** *A. fatua*, *B. graminis* f.sp. *avenae*, resistance

## Abstract

Identifying effective sources of disease resistance is an important aspect of an effective plant protection strategy. Wild species related to cultivars constitute a rich reservoir of resistance genes. Studies conducted in oat have shown that wild species are donors of resistance genes to crown and stem rust, powdery mildew or fusarium head blight. The aim of the present study was to prove whether *A. fatua* could be a source of effective resistance genes to powdery mildew. This species is widespread all over the world due to its very good adaptability and can be regarded as a potential source of resistance to fungal diseases, including powdery mildew. The conducted research has shown that *A. fatua* is a species with a low level of resistance to powdery mildew when compared to other wild species of the genus *Avena* L. A total of 251 accessions were evaluated, and only 23 were identified as resistant to the individual isolates used in the host-pathogen tests. It follows that resistance to powdery mildew is not common among wild *Avena* species, and its good environmental adaptation is not associated to resistance to powdery mildew.

## 1. Introduction

Common oat (*Avena sativa* L.) is an important cereal crop grown worldwide. It withstands and even thrives in poor and adverse conditions that may prove a challenge for other cereal crops cultivated mainly as a forage across the globe [1]. Due to its rich composition of proteins, fats, as well as macro and micronutrients, oat is used not only in animal nutrition but also in human nutrition as a functional food [2,3]. Plant diseases are one of the major constraints in global oat production. Among many diseases affecting oat, fungal diseases such as crown rust, stem rust, powdery mildew and fusarium head blight are the most damaging, deteriorating the yield as well as the quality of the grain and forage [1,4]. Powdery mildew caused by *Blumeria graminis* f.sp. *avenae* appears every year in many part of the world, with varying degrees of severity, causing yield losses ranging from 10 to 39% [5,6]. The appearance of the disease causes a reduction in kernel mass and a significant change in the protein content [7]. Since oat is often grown as a low-input crop on less productive areas, breeding cultivars with a genetically controlled resistance is the most economical and environmentally safe method of controlling fungal diseases in this plant. 

It is known that wild relatives of cultivated crops, which are well adapted to changing environmental conditions, are valuable sources of resistance genes [8,9,10,11,12,13]. Furthermore, wild species belonging to the genus *Avena* L. are a rich source of disease resistance [14], as are several other crop wild relatives (CWR). In fact, CWRs are wild species closely related to crops and constitute potential sources of important traits (such as pest or disease resistance), as well as yield improvement and/or its stability [15]. They are a critical component of plant genetic resources for food and agriculture (PGRFA), although they have been neglected due to in situ and ex situ conservation purposes [16,17]. The identification of wild forms with effective resistance genes is an important step in the process of obtaining cultivars adapted to specific environmental conditions, including those resistant to fungal pathogens present in a specific area. It is necessary to constantly search for effective sources of resistance due to the constant climate changes and rapid adaptation of pathogens to new environmental conditions [18,19,20,21]. Research on the use of wild oat species increasing resistance to fungal diseases has been conducted for many years and allowed one to identify many sources of resistance in diploid, tetraploid and hexaploid species [1].

The resistance gene transfer from wild to cultivated oat is often hindered by sterility barriers [22]. That is why wild species that share all genomes with cultivated oat are the best donors of valuable traits [1,7,23,24,25]; and *A. fatua* L. is one of these species [1,26,27]. It is a wild oat that is widespread all over the world, growing in various climatic zones from the tropics to the polar circle. *A. fatua* is considered to be one of the worst annual weeds in temperate zone cereals [28,29], causing enormous problems in agriculture [30,31,32,33]. Loskutov and Rines [1] have concluded that this species is a source of resistance to various oat diseases. Many years of research have shown that *A. fatua* genotypes can be a source of barley yellow dwarf virus (BYDV) resistance [34,35]. Burdon et al. [36] and Sebesta and Kuhn [37] indicated *A. fatua* as a potential source of crown rust resistance genes, an Fray [38] showed that *A. fatua* genotypes showed resistance to stem rust. This species was also reported as resistant to fusarium head blight [39]. Herrmann and Roderick [24] identified *A. fatua* genotypes as a source of resistance to powdery mildew. All these studies have demonstrated that this species is very interesting and should be studied in more detail. Therefore, the aim of the current study was to determine the possibility of using *A. fatua* genotypes as a source of effective genes against powdery mildew in oat.

## 2. Materials and Methods

**Figure 1:**
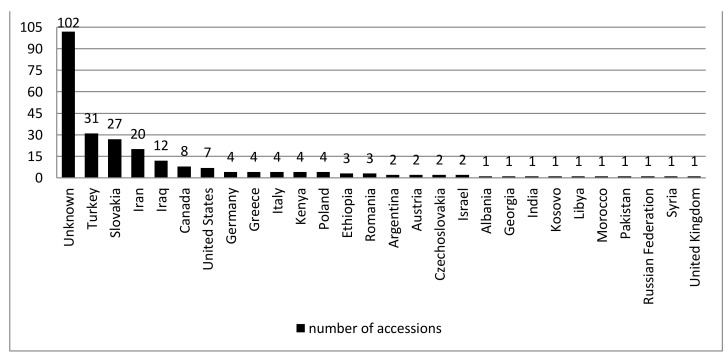
Origin of the analyzed *A. fatua* accessions.

The study objects were 251 *A. fatua* accessions from different parts of the world (Figure 1). All genotypes were obtained from the two gene banks: Plant Gene Resources of Canada, Saskatoon, Canada and Leibniz Institute of Plant Genetics and Crop Plant Research, Gatersleben, Germany.

The resistance of *A. fatua* genotypes was analyzed in host-pathogen tests [40] based on six *Blumeria graminis* f.sp. *avenae* isolates. Single spore isolates were obtained from a population collected in Poland in 2014–2019. The virulence of each isolate was tested on a set of 12 differential oat lines and cultivars with different powdery mildew resistance genes: Jumbo—*Pm1*, Cc3678—*Pm2*, Mostyn—*Pm3*, AV1860—*Pm4*, Am27—*Pm5*, Bruno—*Pm6*, APR122—*Pm7* Rollo—*Pm3+8,* AVE2406—*Pm9*, AVE2925—*Pm10*, CN113536—*Pm11* and Fuchs susceptible to powdery mildew (Table 1). *A. fatua* grains were seeded in plug trays filled with universal substrate and were germinated. After ten days, leaf fragments of the analyzed genotypes were placed on 12-well culture plates with benzimidazole agar (6 g of agar per 1 L of water and 35 mg/L). Leaf fragments of the susceptible cultivar Fuchs were placed into the first and last well of each dish. Plates with leaf fragments were inoculated in an inoculation tower with approximately 500–700 *B. graminis* f sp. *avenae* spores per 1 cm^2^. Subsequently, the plates were incubated in a growing chamber at 17 °C and an illuminance of 4 kLx.

The leaf infection was assessed ten days after inoculation according to a modified 4-point Mains scale [41]. The reactions to the isolates were grouped into three classes: R (resistant)—from 0 to 20% affected leaf area relative to Fuchs, I (intermediate) from 20 to 50%, and S (susceptible)—more than 50% affected leaf area. All tests were performed in three replications to confirm the response of the tested accessions to *B. graminis* f.sp *avenae* isolates. When the genotype response to the applied isolate was different in replications, the highest score was included in the analysis.

## 3. Results

All *A. fatua* accessions were tested using six single spore *B. graminis* f.sp. *avenae* isolates. The isolates selected for the study were characterized by a different level of virulence in relation to the set of control genotypes. The isolates were appropriately selected to ascertain the presence of new sources of resistance in the analyzed genotypes. Based on the infection patterns, isolates were able to distinguish lines with *Pm1*, *Pm3*, *Pm6*, *Pm3* + 8, *Pm9*, *Pm10* and *Pm11* genes (Table 1). All isolates were avirulent to the lines with *Pm2*, *Pm4*, *Pm5* and *Pm7* genes, which confirmed their high efficiency against powdery mildew. 

The resistance reactions of the tested *A. fatua* genotypes varied and ranged from resistant (R) to susceptible (S) (Table 2). Among the 251 tested accessions, 228 showed a susceptible reaction to all *B. graminis* f.sp. *avenae* isolates. Thirteen *A. fatua* accessions showed an intermediate reaction to one or two isolates, and 10 showed resistant reactions to at least one of six *B. graminis* f.sp. *avenae* isolates used in the host-pathogen tests (Supplementary Table S1). None of the tested genotypes was resistant to three or more *B. graminis* f.sp. *avenae* isolates.

Based on the reaction to *B. graminis* f.sp *avenae* isolates, 12 infection patterns were determined among 251 *A. fatua* accessions (Table 3). The obtained infection patterns were compared with those of differential genotypes. The patterns identified in four accessions (Table 3) corresponded to the pattern represented by the cultivar Jumbo carrying the *Pm1* gene. Among the tested *A. fatua* accessions, CN 25174 represented a pattern corresponding to the *Pm3* gene. The rest of the identified patterns in the tested genotypes were unique and did not match the patterns represented by differentials with known *Pm* genes.

Genotypes resistant to at least one of the isolates, according to information obtained from the gene banks, were from Turkey, Iraq, Morocco and Ethiopia, while two were of unknown origin. Genotypes exhibiting a moderate response to at least one *Bga* isolate came from Central and Eastern Europe (Poland, Slovakia, Georgia), while four of them were of unknown origin.

## 4. Discussion

The best way to control disease is to grow genetically resistant cultivars. The number of effective genes against specific pathogens determines the effectiveness of the used protection strategies. So far, 11 resistance genes to powdery mildew have been identified in oat. These genes were introduced into the cultivars from *A. sterilis* L. (*Pm1, Pm3, Pm8* and *Pm11*), *A. byzantina* L. (*Pm6, Pm9, Pm10) A. hirula* L. (*Pm2*), *A. barbata* L. (*Pm4*), *A. macrostachya* L. (*Pm5*) and *A. eriantha* L. (*Pm7*) [5,42,43,44,45,46]. However, none of the described resistance genes are derived from *A. fatua*; therefore, in the present study we attempted to determine whether *A. fatua* could be used as a source of effective resistance genes against powdery mildew in oat. The obtained results allowed for the identification of resistant genotypes; however, none of them were characterized by a broad spectrum of resistance to six different isolates used in the experiment. Okoń et al. [47] analyzed the resistance of selected genotypes of the genus *Avena*. Among others, they analyzed 11 genotypes of *A. fatua*. Tests performed on a set of three *B. graminis* f.sp *avenae* isolates showed that these genotypes were highly susceptible to powdery mildew but showed a moderate level of resistance to one of the isolates. Genotypes AVE 270, AVE1322 and AVE2804 were also tested in the present work, and two of them (AVE 270 and AVE2804) were susceptible to the applied *B. graminis* f.sp *avenae* isolates. Genotype AVE1322 showed resistance to two isolates and moderate resistance to one of them, while in the study by Okoń et al. [47] it was moderately resistant to one isolate used in the tests. These results indicate that this genotype may be of interest in programs aimed at increasing resistance to powdery mildew.

Herrmann and Roderick [24] characterized the powdery mildew resistance of wild species of the genus *Avena*. As a result, they identified a number of resistant genotypes, including *A. fatua* (AVE1981 and AVE 2032). The same genotypes were analyzed in the present work. They were susceptible to all *B. graminis* f.sp *avenae* isolates used in this work. The varied response of the studied genotypes to infection was largely related to changes in virulence occurring in the pathogen population but could also indicate that resistance tests with one isolate did not reflect the actual level of resistance of the genotype. The results of these experiments have indicated that works aimed at identifying effective sources of resistance should be carried out on the basis of a set of isolates with different virulence levels. Similar conclusions were drawn by Okoń and Ociepa [48], who characterized sources of resistance to powdery mildew identified in *A. sterilis* genotypes. Loskutov [49] also drew attention to the fact that effective sources of resistance should be sought among genotypes originating from the area of *Avena* origin, i.e., the Mediterranean region. This thesis was confirmed by Gnanesh et al. [20] and Sebesta et al. [50], who identified *A. sterils* genotypes resistant to crown rust from this region. Okoń et al. [51] identified effective sources of resistance to powdery mildew among *A. sterilis* genotypes from the Mediterranean region. The results obtained in the current work have also confirmed the thesis that the Mediterranean region is the most suitable for searching for effective sources of resistance.

The Mediterranean region, due to its good climatic conditions, favors the development of fungal pathogens, which, thanks to mild winters, can undergo a full development cycle and create new, sometimes more virulent, pathotypes [52]. Such conditions are conducive to the strong pressure of pathogens on plants [53]. Sebesta et al. (1997) noted a greater diversity of pathogens in this region, explaining it as a result of good wintering in the uredial stage. Moreover, the results obtained in our experiments also indicated that genotypes adapted to local conditions could also be valuable in increasing disease resistance. Many studies have shown that landraces can be a valuable source of resistance to pathogens. Well adapted to local conditions, they allow increasing resistance to races appearing in a specific area [12,54,55,56]. The *B. graminis* f.sp *avenae* isolates analyzed in the study originated from pathogen populations collected in various regions of Poland. This allowed for the identification of *A. fatua* accessions from Central Europe showing moderate resistance to selected isolates.

High and moderate resistance to single isolates enable the use of selected genotypes to increase the level of cultivars’ resistance only in a specific area or in combination with other resistance genes, so that protection is maintained for a longer period of time. Partial resistance, usually conferred polygenically, does not completely prevent infection but reduces the pustule size and numbers of produced spores and extends the latency period of pustule development [57]. Partial resistance may be more durable than specific resistance because there is less selection pressure on the pathogen, and it therefore slows the evolution of virulence [58]. Although no completely resistant forms to powdery mildew have been identified among the tested genotypes, the results are of great importance. They demonstrate that disease resistance is not a universal and widespread feature among wild species of the genus *Avena* and that the identification of new, effective genes is a difficult and labor-intensive process requiring the testing of a large number of genotypes with a set of isolates with different virulence.

## Figures and Tables

**Table 1:** Virulence of *B. graminis* f.sp. *avenae* isolates chosen for testing *A. fatua* genotypes.

*B. graminis* f.sp. *avenae* Isolates	Control Lines and Cultivars												
	Jumbo Pm1	Cc3678 Pm2	Mostyn Pm3	Av1860 Pm4	Am 27 Pm5	Bruno Pm6	APR122 Pm7	Canyon Pm7	Rollo Pm3+8	Pm9	Pm10	Pm11	Fuchs
Białka 2014	R	R	S	R	R	S	R	S	I	I	S	R	S
Polanowice 6 2018	S	R	R	R	R	S	R	R	R	I	I	R	S
Danko 2 2019	S	R	R	R	R	S	R	R	I	R	I	S	S
Strzelce 1 2019	S	R	S	R	R	S	R	I	S	R	I	R	S
Felin 2 2018	S	R	R	R	R	S	R	I	R	R	I	R	S
Danko 1 2019	S	R	S	R	R	R	R	I	S	R	R	R	S

R = resistant, I—intermediate, and S = susceptible.

**Table 2:** Number of *A. fatua* accessions resistant (R), intermediate resistant (I) and susceptible (S) to six *B. graminis* f.sp. *avenae* isolates.

*B. graminis* f.sp. *avenae* Isolate	Reaction Type		
	R	I	S
Białka 2014	7	4	240
Danko 2 2019	3	1	247
Strzelce 1 2019	0	2	249
Felin 2 2018	0	13	238
Polanowice 6 2018	1	0	250
Danko 1 2019	0	0	251

**Table 3:** Infection patterns and origin of analyzed *A. fatua* accessions based on the reaction to *B. graminis* f.sp. *avenae* isolates.

Infection Pattern	Accession Numbers	Białka 2014	Danko 2 2019	Strzelce 1/2019	Felin 2/2018	Polanowice 6 /2018	Danko 1 2019	Corresponding Phenotype of *Pm* Genes
1	CN 3498 (U), CN 3512 (U), CN 19401 (IRQ), CN 19415 (IRQ)	R	S	S	S	S	S	*Pm1*
2	CN 25171 (TUR), CN 25176 (TUR)	S	R	S	S	S	S	
3	CN 4248 (TUR), AVE 1318 (MAR), AVE 1322 (ETH)	R	S	S	I	S	S	
4	CN 25174 (TUR)	S	R	S	S	R	S	*Pm3*
5	CN 3664 (U), AVE 1476 (POL), AVE 2103 (SVK), AVE 2106 (SVK), CN 3585 (U), CN 3356 (U), CN 3351 (U)	S	S	S	I	S	S	
6	AVE 1396 (CSV)	I	S	S	I	S	S	
7	AVE 1431 (SVK)	S	S	I	I	S	S	
8	AVE 1442 (SVK)	I	S	S	S	S	S	
9	AVE 1760 (SVK)	I	I	S	S	S	S	
10	AVE 2100 (SVK)	S	S	S	I	S	S	
11	AVE 2679 (GEO)	I	S	I	S	S	S	
12	remaining *A. fatua* genotypes	S	S	S	S	S	S	

U—unknown, IRQ—Iraq, TUR—Turkey, MAR—Morocco, POL—Poland, SVK—Slovakia, CSV—Czechoslovakia, GEO—Georgia.

## Data Availability

Data are available upon request from the corresponding authors.

## Supplementary Materials

The following are available online at https://www.mdpi.com/2223-7747/10/3/560/s1, Supplementary Table S1. Response of A. fatua genotypes to selected B. graminis f.sp. avenae isolates.

## References

[1] Loskutov I.G., Rines H.W. (2011). Avena. Wild Crop Relatives: Genomic and Breeding Resources.

[2] Gorash A., Armonienė R., Mitchell Fetch J., Liatukas Ž., Danytė V. (2017). Aspects in oat breeding: Nutrition quality, nakedness and disease resistance, challenges and perspectives. Ann. Appl. Biol..

[3] Sterna V., Zute S., Brunava L. (2016). Oat Grain Composition and its Nutrition Benefice. Agric. Agric. Sci. Procedia.

[4] Cabral A.L., Gnanesh B.N., Fetch J.M., McCartney C., Fetch T., Park R.F., Menzies J.G., McCallum B., Nanaiah G.K., Goyal A. (2014). Oat Fungal Diseases and the Application of Molecular Marker Technology for Their Control.

[5] Lawes D.A., Hayes J.D. (1965). The effect of mildew (*Erysiphe graminis* f. sp. *avenae*) on spring oats. Plant Pathol..

[6] Jones I.T., Roderick H.W., Clifford B.C. (1987). The integration of host resistance with fungicides in the control of oat powdery mildew. Ann. Appl. Biol..

[7] Roderick H.W., Jones E.R.L., Šebesta J., Sebesta J. (2000). Resistance to oat powdery mildew in Britain and Europe: A review. Ann. Appl. Biol..

[8] McIntosh R.A., Hart G.E., Devos K.M., Gale M.D., Rogers W.J., Slinkard A.E. (1998). Catalogue of gene symbols for wheat. Proceedings of the 9th International Wheat Genetics Symposium.

[9] Shi A.N., Leath S., Murphy J.P. (1998). A major gene for powdery mildew resistance transferred to common wheat from wild einkorn wheat. Phytopathology.

[10] Xu J., Kasha K.J. (1992). Transfer of a dominant gene for powdery mildew resistance and DNA from Hordeum bulbosum into cultivated barley (*H. vulgate*). Theor. Appl. Genet..

[11] Pickering R.A., Hill A.M., Michel M., Timmerman-Vaughan G.M. (1995). The transfer of a powdery mildew resistance gene from *Hordeum bulbosum* L. to barley (*H. vulgare* L.) chromosome 2 (21). Theor. Appl. Genet..

[12] Sánchez-Martín J., Rubiales D., Sillero J.C., Prats E. (2012). Identification and characterization of sources of resistance in *Avena sativa*, *A. byzantinea* and *A. strigosagermplasm* against a pathotype of *Puccinia coronate* f.sp. *avenae* with virulence against the Pc94 resistance gene. Plant Pathol..

[13] Tan M.Y.A., Carson M.L. (2013). Screening Wild Oat Accessions from Morocco for Resistance to Puccinia coronata. Plant Dis..

[14] Loskutov I.G. (1998). The collection of wild oat species of C.I.S. as a source of diversity in agricultural traits. Genet. Resour. Crop Evol..

[15] Perrino E.V., Wagensommer R.P. (2021). Crop Wild Relatives (CWR) Priority in Italy: Distribution, Ecology, In Situ and Ex Situ Conservation and Expected Actions. Sustainability.

[16] Perrino E.V., Perrino P. (2020). Crop wild relatives: Know how past and present to improve future research, conservation and utilization strategies, especially in Italy: A review. Genet. Resour. Crop Evol..

[17] Zair W., Maxted N., Brehm J.M., Amri A. (2021). Ex situ and in situ conservation gap analysis of crop wild relative diversity in the Fertile Crescent of the Middle East. Genet. Resour. Crop Evol..

[18] Hsam S.L.K., Paderina E.V., Gordei S., Zeller F.J. (1998). Genetic studies of powdery mildew resistance in cultivated oat (*Avena sativa* L.) II. Cultivars and breeding lines grown in Northern and Eastern Europe. Hereditas.

[19] Polák J., Bartoš P. (2002). Natural Sources of Plant Disease Resistance and their Importance in the Breeding. Czech J. Genet. Plant Breed..

[20] Gnanesh B.N., Fetch J.M., Zegeye T., McCartney C.A., Fetch T. (2014). Oat. Alien Gene Transfer in Crop Plants.

[21] Nazir N., Bilal S., Bhat K., Shah T., Badri Z., Bhat F., Wani T., Mugal M., Parveen S., Dorjry S. (2018). Effect of Climate Change on Plant Diseases. Artic. Int. J. Curr. Microbiol. Appl. Sci..

[22] Aung T., Thomas H., Jones I.T. (1977). The transfer of the gene for mildew resistance from *Avena barbata* (4x) into the cultivated oat *A. sativa* by an induced translocation. Euphytica.

[23] Sebesta J., Roderick H.W., Chong J., Harder D.E. (1993). The oat line Pc54 as a source of resistance to crown rust, stem rust and powdery mildew in Europe. Euphytica.

[24] Herrmann M., Roderick H.W. (1996). Characterisation of new oat germplasm for resistance to powdery mildew. Euphytica.

[25] Cabral A.L., Park R.F. (2014). Seedling resistance to *Puccinia coronata* f. sp. *avenae* in *Avena strigosa*, *A. barbata* and *A. sativa*. Euphytica.

[26] Vavilov N.I. (1926). The centres of origin of cultivated plants. Appl. Bot. Plant Breed.

[27] Loskutov I.G. (2008). On evolutionary pathways of *Avena* species. Genet. Resour. Crop Evol..

[28] Bajwa A.A., Akhter M.J., Iqbal N., Peerzada A.M., Hanif Z., Manalil S., Hashim S., Ali H.H., Kebaso L., Frimpong D. (2017). Biology and management of *Avena fatua* and *Avena ludoviciana*: Two noxious weed species of agro-ecosystems. Environ. Sci. Pollut. Res..

[29] Beckie H.J., Francis A., Hall L.M. (2012). The Biology of Canadian Weeds. 27. *Avena fatua* L. (updated). Can. J. Plant Sci..

[30] Aibar J., Ochoa M.J., Zaragoza C. (1991). Field emergence of *Avena fatua* L. and *A. sterilis* ssp. *ludoviciana* (Dur.) Nym. in Aragon, Spain. Weed Res..

[31] Harker K.N., O’Donovan J.T., Turkington T.K., Blackshaw R.E., Lupwayi N.Z., Smith E.G., Johnson E.N., Pageau D., Shirtliffe S.J., Gulden R.H. (2016). Diverse Rotations and Optimal Cultural Practices Control Wild Oat (*Avena fatua*). Weed Sci..

[32] Heap I. (2014). Global perspective of herbicide-resistant weeds. Pest Manag. Sci..

[33] Mahajan G., Loura D., Raymont K., Chauhan B.S. (2020). Influence of soil moisture levels on the growth and reproductive behaviour of *Avena fatua* and *Avena ludoviciana*. PLoS ONE.

[34] Rines H.W., Stuthman D.D., Briggle L.W., Youngs V.L., Jedlinski H., Smith D.H., Webster J.A., Rothman P.G. (1980). Collection and Evaluation of *Avena fatua* for Use in Oat Improvement. Crop Sci..

[35] Comeau A. (1984). Barley Yellow Dwarf Virus Resistance in the Genus *Avena*. Euphytica.

[36] Burdon J.J., Oates J.D., Marshall D.R. (1983). Interactions between *Avena* and *Puccinia* Species. I. The Wild Hosts: *Avena barbata* Pott Ex Link, *A. fatua* L. *A. ludoviciana* Durieu. J. Appl. Ecol..

[37] Šebesta J., Kühn F. (1990). *Avena fatua* L. subsp. *fatua v. glabrata* Peterm. subv. *pseudo-basifixa* Thell. as a source of crown rust resistance genes. Euphytica.

[38] Frey K.J. (1991). Genetic Resources of Oats. Use of Plant Introductions in Cultivar Development Part 1.

[39] Gagkaeva T., Gavrilova O.P., Yli-Mattila T., Loskutov I.G. (2013). Sources of resistance to Fusarium head blight in VIR oat collection. Euphytica.

[40] Hsam S.L.K., Peters N., Paderina E.V., Felsenstein F., Oppitz K., Zeller F.J. (1997). Genetic studies of powdery mildew resistance in common oat (*Avena sativa* L.) I. Cultivars and breeding lines grown in Western Europe and North America. Euphytica.

[41] Mains E.B. (1934). Inheritance of resistance to powdery mildew, Erysiphe graminis tritici, in wheat. Phytopathology.

[42] Hayes J.D., Catling W.S. (1963). Physiological specialization in Erysiphe graminis DC. in oats. Nature.

[43] Thomas H., Powell W., Aung T. (1980). Interfering with regular meiotic behaviour in *Avena sativa* as a method of incorporating the gene for mildew resistance from *A. barbata*. Euphytica.

[44] Yu J., Herrmann M. (2006). Inheritance and mapping of a powdery mildew resistance gene introgressed from *Avena macrostachya* in cultivated oat. Theor. Appl. Genet..

[45] Herrmann M.H., Mohler V. (2018). Locating two novel genes for resistance to powdery mildew from *Avena byzantine* in the oat genome. Plant. Breed..

[46] Ociepa T., Okoń S., Nucia A., Leśniowska-Nowak J., Paczos-Grzęda E., Bisaga M. (2020). Molecular identification and chromosomal localization of new powdery mildew resistance gene Pm11 in oat. Theor. Appl. Genet..

[47] Okoń S.M., Chrzastek M., Kowalczyk K., Koroluk A., Chrząstek M., Kowalczyk K., Koroluk A. (2014). Identification of new sources of resistance to powdery mildew in oat. Eur. J. Plant. Pathol..

[48] Okoń S.M., Ociepa T. (2018). Effectiveness of new sources of resistance against oat powdery mildew identified in *A. sterilis*. J. Plant. Dis. Prot..

[49] Loskutov I.G. Using of wild species genetic diversity in plant breeding. Proceedings of the 4th International Crop Science Congress.

[50] Sebesta J., Zwatz B., Roderick H., Corazza L., Manisterski J., Stojanovic S. (2003). Incidence of crown rust and virulence of *Puccinia coronata* cda. f.sp. *avenae* eriks. and the effectiveness of pc genes for resistance in Europe, middle east and north Africa. Arch. Phytopathol. Plant Prot..

[51] Okoń S., Paczos-Grzeda E., Ociepa T., Koroluk A., Sowa S., Kowalczyk K., Chrzą M. (2016). *Avena sterilis* L. Genotypes as a Potential Source of Resistance to Oat Powdery Mildew. Plant. Dis..

[52] Clifford B.C., Welch R.W. (1995). Diseases, pest and disorders of oat. The Oat Crop.

[53] Braun U., Cook R.T.A., Inman A.J., Shin H.D. (2002). The taxonomy of the powdery mildew fungi. The Powdery Mildews: A Comprehensive Treatis.

[54] Li H.B., Zhou M.X., Liu C.J. (2009). A major QTL conferring crown rot resistance in barley and its association with plant height. Theor. Appl. Genet..

[55] Saker M., Adawy S., Smith C.M. (2008). Entomological and genetic variation of cultivated barley (*Hordeum vulgare*) from Egypt. Arch. Phytopathol. Plant. Prot..

[56] Sánchez-Martín J., Rubiales D., Prats E. (2011). Resistance to powdery mildew (*Blumeria graminis* f.sp. *avenae*) in oat seedlings and adult plants. Plant. Pathol..

[57] Portyanko V.A., Hoffman D.L., Lee M., Holland J.B. (2001). A linkage map of hexaploid oat based on grass anchor DNA clones and its relationship to other oat maps. Genome.

[58] Simons M.D. (1972). Polygenic Resistance to Plant Disease and Its Use in Breeding Resistant Cultivars. J. Environ. Qual..

