# Supplementary material for: Is Every Wild Species a Rich Source of Disease Resistance? Avena fatua L.—Potential Donor of Resistance to Powdery Mildew
Source: Plants (Basel). 2021 Mar 16;10(3):560. doi: 10.3390/plants10030560 (PMC8002336; doi:10.3390/plants10030560)
Supplement: Supplementary file 1 [file plants-10-00560-s001.pdf]

Supplementary table 1. Response of *A. fatua* genotypes to selected *B. graminis* f.sp. *avenae* isolates.

Access number	Białka 2014	Danko 2 2019	Strzelce 1/2019	Felin 2/2018	Polanowice 6 /2018	Danko 1 2019	Country of origin	Access number	Białka 2014	Danko 2 2019	Strzelce 1/2019	Felin 2/2018	Polanowice 6 /2018	Danko 1 2019	Country of origin
AVE 1318	R	S	S	I	S	S	Morocco	CN 25129	S	S	S	S	S	S	Turkey
AVE 1322	R	S	S	I	S	S	Ethiopia	CN 25146	S	S	S	S	S	S	Turkey
AVE 1396	I	S	S	I	S	S	Czechoslovakia	CN 25171	S	R	S	S	S	S	Turkey
AVE 1431	S	S	I	I	S	S	Slovakia	CN 25174	S	R	S	S	R	S	Turkey
AVE 1441	S	S	S	S	S	S	Slovakia	CN 25176	S	R	S	S	S	S	Turkey
AVE 1442	I	S	S	S	S	S	Slovakia	CN 25191	S	S	S	S	S	S	Syria
AVE 1476	S	S	S	I	S	S	Poland	CN 30542	S	S	S	S	S	S	United Kingdom
AVE 1515	S	S	S	S	S	S	Czechoslovakia	CN 3214	S	S	S	S	S	S	Unknown
AVE 1516	S	S	S	S	S	S	Slovakia	CN 3215	S	S	S	S	S	S	Unknown
AVE 1565	S	S	S	S	S	S	United States	CN 3218	S	S	S	S	S	S	Unknown
AVE 1566	S	S	S	S	S	S	Canada	CN 3220	S	S	S	S	S	S	Unknown
AVE 161	S	S	S	S	S	S	Turkey	CN 3224	S	S	S	S	S	S	Unknown
AVE 1707	S	S	S	S	S	S	Slovakia	CN 3225	S	S	S	S	S	S	Unknown
AVE 1710	S	S	S	S	S	S	Slovakia	CN 3227	S	S	S	S	S	S	Unknown
AVE 1758	S	S	S	S	S	S	Slovakia	CN 3228	S	S	S	S	S	S	Unknown
AVE 1760	I	I	S	S	S	S	Slovakia	CN 3229	S	S	S	S	S	S	Unknown
AVE 1764	S	S	S	S	S	S	Slovakia	CN 3230	S	S	S	S	S	S	Unknown
AVE 1765	S	S	S	S	S	S	Slovakia	CN 3237	S	S	S	S	S	S	Unknown
AVE 1772	S	S	S	S	S	S	Canada	CN 3238	S	S	S	S	S	S	Unknown
AVE 1773	S	S	S	S	S	S	United States	CN 3239	S	S	S	S	S	S	Unknown
AVE 1780	S	S	S	S	S	S	Poland	CN 3271	S	S	S	S	S	S	Unknown
AVE 191	S	S	S	S	S	S	Greece	CN 3305	S	S	S	S	S	S	Unknown
AVE 1923	S	S	S	S	S	S	Slovakia	CN 3306	S	S	S	S	S	S	Unknown
AVE 1934	S	S	S	S	S	S	Slovakia	CN 3329	S	S	S	S	S	S	Unknown
AVE 1981	S	S	S	S	S	S	Ethiopia	CN 3350	S	S	S	S	S	S	Unknown
AVE 2000	S	S	S	S	S	S	Germany	CN 3351	S	S	S	I	S	S	Unknown
AVE 2003	S	S	S	S	S	S	Slovakia	CN 3353	S	S	S	S	S	S	Unknown
AVE 2032	S	S	S	S	S	S	Poland	CN 3355	S	S	S	S	S	S	Unknown
AVE 2044	S	S	S	S	S	S	Romania	CN 3356	S	S	S	I	S	S	Unknown
AVE 2085	S	S	S	S	S	S	Italy	CN 3383	S	S	S	S	S	S	Unknown
AVE 2090	S	S	S	S	S	S	Slovakia	CN 3396	S	S	S	S	S	S	Unknown
AVE 2095	S	S	S	S	S	S	Slovakia	CN 3398	S	S	S	S	S	S	Unknown
AVE 2096	S	S	S	S	S	S	Slovakia	CN 3399	S	S	S	S	S	S	Unknown
AVE 2098	S	S	S	S	S	S	Slovakia	CN 3403	S	S	S	S	S	S	Unknown
AVE 2100	S	S	S	I	S	S	Slovakia	CN 3421	S	S	S	S	S	S	Unknown
AVE 2102	S	S	S	S	S	S	Slovakia	CN 3422	S	S	S	S	S	S	Unknown
AVE 2103	S	S	S	I	S	S	Slovakia	CN 3431	S	S	S	S	S	S	Unknown
AVE 2106	S	S	S	I	S	S	Slovakia	CN 3433	S	S	S	S	S	S	Unknown
AVE 2108	S	S	S	S	S	S	Slovakia	CN 3438	S	S	S	S	S	S	Unknown
AVE 2109	S	S	S	S	S	S	Slovakia	CN 3442	S	S	S	S	S	S	Unknown

AVE 2110	S	S	S	S	S	S	Slovakia
AVE 2114	S	S	S	S	S	S	Slovakia
AVE 2211	S	S	S	S	S	S	Slovakia
AVE 2407	S	S	S	S	S	S	Libya
AVE 2428	S	S	S	S	S	S	Slovakia
AVE 2649	S	S	S	S	S	S	Italy
AVE 2671	S	S	S	S	S	S	Austria
AVE 2673	S	S	S	S	S	S	Austria
AVE 2679	I	S	I	S	S	S	Georgia
AVE 270	S	S	S	S	S	S	Albania
AVE 2804	S	S	S	S	S	S	Romania
AVE 2805	S	S	S	S	S	S	Romania
AVE 3138	S	S	S	S	S	S	Italy
AVE 3214	S	S	S	S	S	S	Unknown
AVE 3301	S	S	S	S	S	S	Italy
AVE 437	S	S	S	S	S	S	Germany
AVE 438	S	S	S	S	S	S	Greece
AVE 439	S	S	S	S	S	S	Greece
AVE 440	S	S	S	S	S	S	Greece
AVE 4758	S	S	S	S	S	S	Germany
AVE 5087	S	S	S	S	S	S	Poland
AVE 527	S	S	S	S	S	S	Turkey
AVE 725	S	S	S	S	S	S	Germany
AVE 996	S	S	S	S	S	S	Ethiopia
CN 106521	S	S	S	S	S	S	Canadaada
CN 106522	S	S	S	S	S	S	United States
CN 106524	S	S	S	S	S	S	Canadaada
CN 106525	S	S	S	S	S	S	Unknown
CN 106526	S	S	S	S	S	S	Canadaada
CN 106529	S	S	S	S	S	S	Unknown
CN 106531	S	S	S	S	S	S	Canadaada
CN 106532	S	S	S	S	S	S	Canadaada
CN 19396	S	S	S	S	S	S	Iraq
CN 19399	S	S	S	S	S	S	Iraq
CN 19400	S	S	S	S	S	S	Iraq
CN 19401	R	S	S	S	S	S	Iraq
CN 19403	S	S	S	S	S	S	Iraq
CN 19408	S	S	S	S	S	S	Iraq
CN 19410	S	S	S	S	S	S	Iraq
CN 19414	S	S	S	S	S	S	Iraq
CN 19415	R	S	S	S	S	S	Iraq
CN 19419	S	S	S	S	S	S	Iraq
CN 19420	S	S	S	S	S	S	Iraq
CN 19435	S	S	S	S	S	S	Iraq
CN 21195	S	S	S	S	S	S	Iran
CN 21197	S	S	S	S	S	S	Iran
CN 21201	S	S	S	S	S	S	Iran
CN 21204	S	S	S	S	S	S	Iran
CN 3443	S	S	S	S	S	S	Unknown
CN 3452	S	S	S	S	S	S	Unknown
CN 3453	S	S	S	S	S	S	Unknown
CN 3476	S	S	S	S	S	S	Unknown
CN 3477	S	S	S	S	S	S	Unknown
CN 3486	S	S	S	S	S	S	Unknown
CN 3488	S	S	S	S	S	S	Unknown
CN 3489	S	S	S	S	S	S	Unknown
CN 3497	S	S	S	S	S	S	Unknown
CN 3498	R	S	S	S	S	S	Unknown
CN 3499	S	S	S	S	S	S	Unknown
CN 3502	S	S	S	S	S	S	Unknown
CN 3503	S	S	S	S	S	S	Unknown
CN 3504	S	S	S	S	S	S	Unknown
CN 3507	S	S	S	S	S	S	Unknown
CN 3512	R	S	S	S	S	S	Unknown
CN 3515	S	S	S	S	S	S	Unknown
CN 3517	S	S	S	S	S	S	Unknown
CN 3518	S	S	S	S	S	S	Unknown
CN 3519	S	S	S	S	S	S	Unknown
CN 3520	S	S	S	S	S	S	Unknown
CN 3521	S	S	S	S	S	S	Unknown
CN 3522	S	S	S	S	S	S	Unknown
CN 3523	S	S	S	S	S	S	Unknown
CN 3524	S	S	S	S	S	S	Unknown
CN 3525	S	S	S	S	S	S	Unknown
CN 3530	S	S	S	S	S	S	Unknown
CN 3531	S	S	S	S	S	S	Unknown
CN 3533	S	S	S	S	S	S	Unknown
CN 3534	S	S	S	S	S	S	Unknown
CN 3535	S	S	S	S	S	S	Unknown
CN 3536	S	S	S	S	S	S	Unknown
CN 3541	S	S	S	S	S	S	Unknown
CN 3548	S	S	S	S	S	S	Unknown
CN 3549	S	S	S	S	S	S	Unknown
CN 3550	S	S	S	S	S	S	Unknown
CN 3560	S	S	S	S	S	S	Unknown
CN 3565	S	S	S	S	S	S	Unknown
CN 3566	S	S	S	S	S	S	Unknown
CN 3567	S	S	S	S	S	S	Unknown
CN 3571	S	S	S	S	S	S	Unknown
CN 3572	S	S	S	S	S	S	Unknown
CN 3576	S	S	S	S	S	S	Unknown
CN 3579	S	S	S	S	S	S	Unknown
CN 3582	S	S	S	S	S	S	Unknown
CN 3583	S	S	S	S	S	S	Unknown
CN 3584	S	S	S	S	S	S	Unknown
CN 3585	S	S	S	I	S	S	Unknown

CN 21208	S	S	S	S	S	S	Iran
CN 21213	S	S	S	S	S	S	Iran
CN 21229	S	S	S	S	S	S	Iran
CN 21241	S	S	S	S	S	S	Iran
CN 21242	S	S	S	S	S	S	Iran
CN 21244	S	S	S	S	S	S	Iran
CN 21245	S	S	S	S	S	S	Iran
CN 21246	S	S	S	S	S	S	Iran
CN 21247	S	S	S	S	S	S	Iran
CN 21248	S	S	S	S	S	S	Iran
CN 21251	S	S	S	S	S	S	Iran
CN 21258	S	S	S	S	S	S	Iran
CN 21259	S	S	S	S	S	S	Iran
CN 21260	S	S	S	S	S	S	Iran
CN 21261	S	S	S	S	S	S	Iran
CN 21263	S	S	S	S	S	S	Iran
CN 21278	S	S	S	S	S	S	Turkey
CN 21281	S	S	S	S	S	S	Turkey
CN 21282	S	S	S	S	S	S	Turkey
CN 21284	S	S	S	S	S	S	Turkey
CN 21285	S	S	S	S	S	S	Turkey
CN 21288	S	S	S	S	S	S	Turkey
CN 21290	S	S	S	S	S	S	Turkey
CN 21292	S	S	S	S	S	S	Turkey
CN 21293	S	S	S	S	S	S	Turkey
CN 21294	S	S	S	S	S	S	Turkey
CN 21297	S	S	S	S	S	S	Turkey
CN 21299	S	S	S	S	S	S	Turkey
CN 21301	S	S	S	S	S	S	Turkey
CN 22550	S	S	S	S	S	S	Turkey
CN 22552	S	S	S	S	S	S	Turkey
CN 22575	S	S	S	S	S	S	Turkey
CN 22607	S	S	S	S	S	S	Kenya
CN 22613	S	S	S	S	S	S	Kenya
CN 22616	S	S	S	S	S	S	Kenya
CN 22617	S	S	S	S	S	S	Kenya
CN 24167	S	S	S	S	S	S	Israel
CN 24240	S	S	S	S	S	S	Israel
CN 3588	S	S	S	S	S	S	Unknown
CN 3590	S	S	S	S	S	S	Unknown
CN 3605	S	S	S	S	S	S	Unknown
CN 3609	S	S	S	S	S	S	Unknown
CN 3610	S	S	S	S	S	S	Unknown
CN 3627	S	S	S	S	S	S	Unknown
CN 3628	S	S	S	S	S	S	Unknown
CN 3630	S	S	S	S	S	S	Unknown
CN 3631	S	S	S	S	S	S	Unknown
CN 3639	S	S	S	S	S	S	Unknown
CN 3664	S	S	S	I	S	S	Unknown
CN 3666	S	S	S	S	S	S	Unknown
CN 3667	S	S	S	S	S	S	Unknown
CN 3672	S	S	S	S	S	S	Unknown
CN 3676	S	S	S	S	S	S	Unknown
CN 3677	S	S	S	S	S	S	Unknown
CN 3680	S	S	S	S	S	S	Unknown
CN 3681	S	S	S	S	S	S	Unknown
CN 42402	S	S	S	S	S	S	United States
CN 4248	R	S	S	I	S	S	Turkey
CN 4261	S	S	S	S	S	S	Turkey
CN 4262	S	S	S	S	S	S	Turkey
CN 4264	S	S	S	S	S	S	Turkey
CN 4265	S	S	S	S	S	S	Turkey
CN 4267	S	S	S	S	S	S	Turkey
CN 4324	S	S	S	S	S	S	Turkey
CN 4336	S	S	S	S	S	S	Turkey
CN 53502	S	S	S	S	S	S	United States
CN 53503	S	S	S	S	S	S	Russian Federation
CN 53861	S	S	S	S	S	S	Canadaada
CN 54879	S	S	S	S	S	S	Argentina
CN 54881	S	S	S	S	S	S	Argentina
CN 55036	S	S	S	S	S	S	United States
CN 55039	S	S	S	S	S	S	United States
CN 64060	S	S	S	S	S	S	Pakistan
CN 72438	S	S	S	S	S	S	Kosovo
CN 88926	S	S	S	S	S	S	India

CN= Plant Gene Resources of Canada.
AVE= Leibniz Institute of Plant Genetics and Crop Plant Research.
R = resistant, I – intermediate, and S = susceptible.
